# A Systematic Review of Perennial Staple Crops Literature Using Topic Modeling and Bibliometric Analysis

**DOI:** 10.1371/journal.pone.0155788

**Published:** 2016-05-23

**Authors:** Daniel A. Kane, Paul Rogé, Sieglinde S. Snapp

**Affiliations:** Dept. of Plant, Soil, and Microbial Sciences, Michigan State University, East Lansing, MI, United States of America; Università Politecnica delle Marche, ITALY

## Abstract

Research on perennial staple crops has increased in the past ten years due to their potential to improve ecosystem services in agricultural systems. However, multiple past breeding efforts as well as research on traditional ratoon systems mean there is already a broad body of literature on perennial crops. In this review, we compare the development of research on perennial staple crops, including wheat, rice, rye, sorghum, and pigeon pea. We utilized the advanced search capabilities of Web of Science, Scopus, ScienceDirect, and Agricola to gather a library of 914 articles published from 1930 to the present. We analyzed the metadata in the entire library and in collections of literature on each crop to understand trends in research and publishing. In addition, we applied topic modeling to the article abstracts, a type of text analysis that identifies frequently co-occurring terms and latent topics. We found: 1.) Research on perennials is increasing overall, but individual crops have each seen periods of heightened interest and research activity; 2.) Specialist journals play an important role in supporting early research efforts. Research often begins within communities of specialists or breeders for the individual crop before transitioning to a more general scientific audience; 3.) Existing perennial agricultural systems and their domesticated crop material, such as ratoon rice systems, can provide a useful foundation for breeding efforts, accelerating the development of truly perennial crops and farming systems; 4.) Primary research is lacking for crops that are produced on a smaller scale globally, such as pigeon pea and sorghum, and on the ecosystem service benefits of perennial agricultural systems.

## 1. Introduction

The breeding of perennial staple crops, such as perennial wheat, has emerged as a novel approach to sustainable agriculture and food systems. Perennial crops could reduce fuel dependency and better protect soil quality compared to conventional production of annual crops [1,2]. In addition to ecological benefits, some argue they could better protect human health by reducing chemical input requirements, provide additional economic opportunities for farmers, and help some vulnerable communities achieve food security [3,4].

This notion has stimulated widespread interest amongst researchers and farmers alike [5]. Yet, the development of perennial forms of crops is not entirely novel. Humans have a long history of collecting the seed from wild perennial grains, such as Kreb, a complex of perennial and annual grass species that has been collected for food in the area near Lake Chad for several centuries [6]. Moreover, farmers in many parts of the world have utilized a technique known as ratooning, wherein crops are cut at the end of a growing season and allowed to regrow into the next, to produce multiple successive crops from perennial legumes such as pigeon pea (*Cajanus cajan*) and cereals such as rice (*Oryza sativa*) and sorghum (*Sorghum bicolor*), which are more commonly used as annuals [7–9]. Hill [7] even suggests that there is archaeological and historical evidence that in Southeast Asia and China rice was first cultivated as a perennial before being domesticated as an annual. In the past century and a half, breeders and agricultural researchers have attempted to develop crops for use as perennials. Several literature reviews have summarized the history of perennial crops research and the conclusions of various projects. Wagoner [10] summarized early attempts to breed perennial wheat by wide hybridization with grass species in the former Soviet Union. Those grain-producing perennial genotypes developed from distant hybridization were frequently sterile and had problems with lodging and winter survival, but they did provide useful genetic material for future breeding efforts. In a series of more recent articles, researchers affiliated with the Land Institute (Salina, KS), an organization with current perennial crop breeding programs, summarize their more recent efforts. Efforts to breed perennial wheat and wheatgrass through both direct domestication and wide hybridization have produced some promising cultivars capable of surviving and producing grain yields every year, but yields decrease after the first season, meaning an extended selection process to improve yield components is necessary [9,11,12]. Interspecific hybridization of *Sorghum bicolor* and *S*. *halpense* created several sterile offspring, but a handful of lines were picked from this initial effort for further development and use in the field [11–13].

DeHaan, et al. [11] and Pimentel, et al. [9] note that promising work on developing perennial upland rice through interspecific hybridization of perennial and annual *Oryza spp*. had been done by the International Rice Research Institute (IRRI) but was abandoned due to changing research priorities. More recently, the Yunnan Academy of Agricultural Sciences has successfully revived these efforts [14,15].

While these reviews provide some information on the history of perennial crop research, their focus is primarily on the potential of perennial crops and theoretical approaches to breeding rather than providing a detailed analysis of existing literature. A review that evaluates the publication histories of different perennial crops and ratooning systems is missing from the literature. Such a systematic review will help identify gaps in the literature by comparing the successes and failures associated with developing perennial staple crops as well as how research trajectories affect the current status of each crop analyzed.

Our objective was to assemble a comprehensive library of literature on principal staple crops for which perennial forms are under development in order to study the progression of perennial crops research over time. We excluded crops that have perennial forms of potential interest, such as *Phaseolus coccineus*, as we were more interested in how previous literature might explain the current status of perennial lines that are actively being developed. After an initial analysis of bibliographic metadata, we applied a topic modeling approach to identify topics in the perennial crops literature. We looked for changes in the topics across the perennial crops library, as well as within the collections of literature for each crop of interest. Topic modeling is a novel approach to objectively evaluating the development of a research area by identifying emergent themes and topics in a body of literature via statistical algorithms.

## 2. Methods

We analyzed the scientific literature on a total of five crops: pigeon pea, rice, rye, sorghum, and wheat. These crops have viable perennial forms that are under active development and could have the potential to be used in both commercial and smallholder cropping systems. In addition, we analyzed the literature containing the keywords “perennial grain” to capture more general reviews on the concept of perennial cropping systems. The term “perennial grains” is frequently used to refer to the concept after seminal work from researchers associated with the Land Institute.

### 2.2. Literature searches and bibliometric analysis

We began by assembling a comprehensive library on the perennial forms of the target crops using the advanced search capabilities of four databases to ensure adequate coverage of the scientific literature: Web of Science [16], Scopus [17], ScienceDirect [18], and AGRICOLA [19]. We conducted the searches on June 3, 2015 and retrieved 3026 records, including duplicates. The search results were limited to journal articles, but no constraints were placed on date of publication.

Searches were composed of two inclusion terms linked by a proximity operator Table 1. The first inclusion term was a modifier word or phrase that limited results to journal articles that pertained specifically to the perennial forms of the crop, while the second inclusion term was either the common or scientific genus name of the crop of interest (example: “perennial W/1 wheat”).

**Table 1:** Table detailing the construction of searches based on the combination of two inclusion terms and a proximity operator for the inclusion terms. In one case an exclusion term was used to avoid extraneous literature.

Inclusion Term #1	Inclusion Term #2	Proximity Operator	Exclusion term
perennial	wheat, grain, triticum, secale, pigeon pea, cajanus, rice, oryza	1	
perennial	rye	1	ryegrass
long duration	pigeon pea, cajanus	1	
ratoon	sorghum, pigeon pea, cajanus, rice. oryza	5	

For the most part, the proximity operator was set to “1,” which meant that the two inclusion terms were within one word of each other. This approach was less restrictive than searching for exact phrases (i.e. “perennial wheat”). But it did better at reducing false hits than the "AND" Boolean operator, such as instances where inclusion terms might appear in unrelated parts of the text. The proximity operator was increased to “5” for the inclusion term “ratoon.” As a cultural practice, the term ratooning retains relevance even if it is separated by a greater number of words from the crop of interest. The term “ratoon” was paired with the terms “rice” and “sorghum,” as ratooning is a common practice with these crops. The searches for rye were further refined to exclude results that referenced perennial forage and weed perennial ryegrass. This conservative approach may have inadvertently excluded some relevant literature on perennial rye.

In choosing our search criteria, we also made certain inadvertent and intentional omissions. Searches were centered on American English to the neglect of foreign languages and other English dialects. Potentially useful terms like “perennial cereals,” “rhizome,” and “stolon” would have likely contributed additional material of relevance, as would have searches for emerging perennial crops like wild rice (*Zizania* spp.) and kernza (*Thinopyrum intermedium*). However, as we were primarily interested in examining how historical literature potentially influenced current status, we chose to exclude emerging crops that did not have a sufficient development history, as well as extraneous terms that may have yielded interesting, relevant literature but were not directly related to the crops of interest.

Following the searches, we imported search results into the citation manager Zotero [20] and tagged bibliographic records with both the name of the database and search that retrieved it. We then merged duplicate entries using Zotero’s duplicate merge tool [21]. Merged entries retained the tags that were applied to the original bibliographic records. Finally, a manual merge was conducted on a small number of entries that had escaped detection. Book chapters and author indices were also removed. We stripped abstracts of copyright information and headings, and located missing abstracts using the Digital Object Identifier [22] and Google Scholar [23].

**Fig 1:**
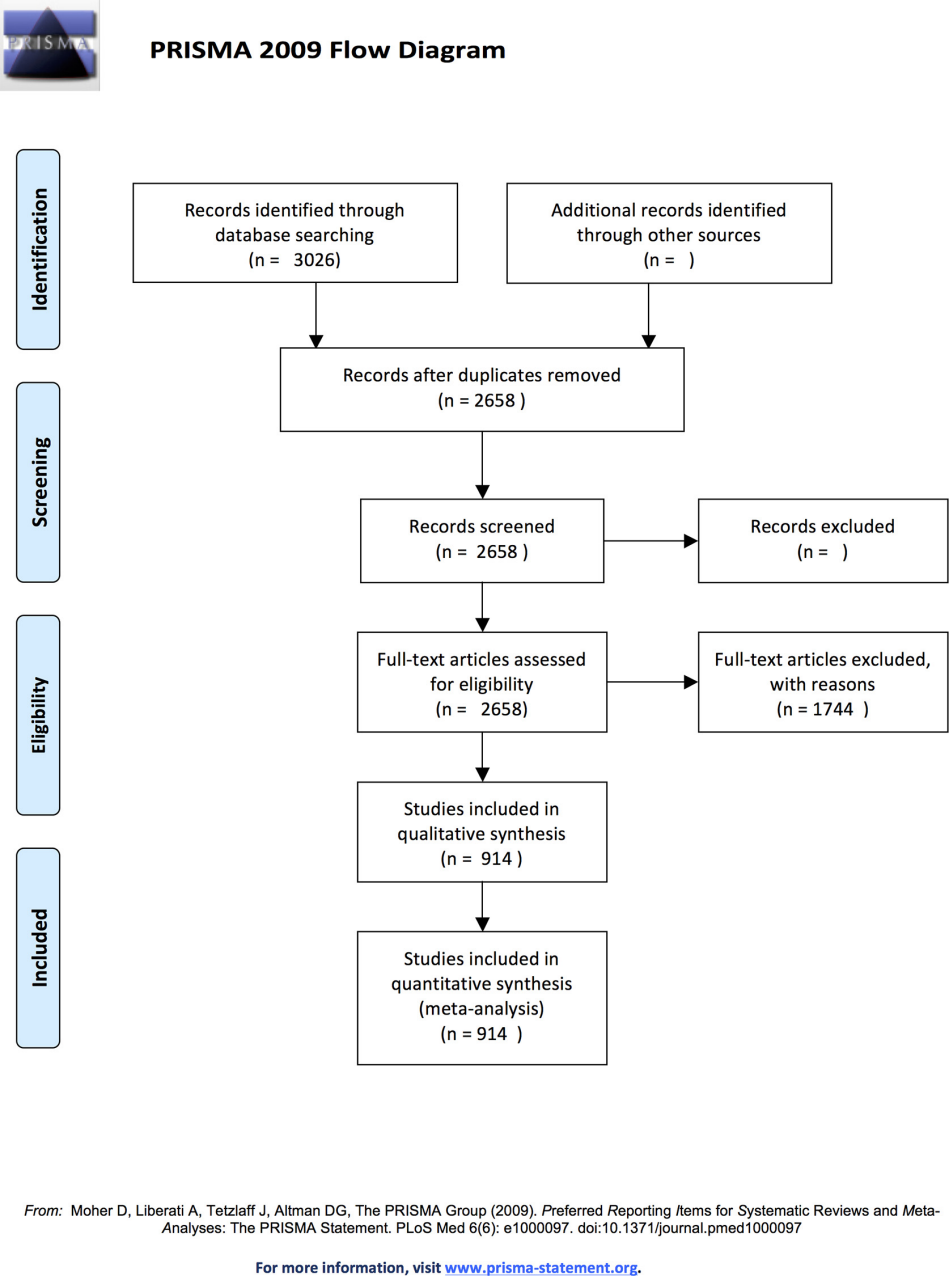
PRISMA flow diagram detailing screening process of articles included in analysis.

Our search criteria retrieved a total of 2658 unique articles. But after detailed review by a single reviewer, only 914 were deemed relevant Fig 1. Articles spanned a variety of topics and research areas beyond perennial crops, necessitating the creation of detailed criteria for exclusion or inclusion. Articles specifically addressing the breeding or use of perennial crops for food production were retained, while articles that dealt only with their use for forage, silage, or bioenergy were eliminated. Such articles may be useful to researchers interested in perennial crops, however we chose to exclude them since we were interested in literature explicitly examining the development and use of perennial crops to produce food for human consumption. Searches using the “ratoon” search term returned several papers about crops that were harvested, ratooned, and harvested for a second crop within the same 12 months. We chose to retain these articles because they included information highly applicable to the development of truly perennial crops and cropping systems.

Many searches returned articles on perennial wild relatives of the crops of interest. Since perennial wild relatives are often used as the genetic source material for developing perennial crops, the relevance of these articles were particularly difficult to discern. We retained articles on the traditional usage of wild relatives of domesticated crops since these systems present interesting analogues. Articles on the genetics of wild relatives that dealt with sequencing the entire genome or investigated the genetic basis of specific plant traits that might make them useful perennials (i.e. rhizomatousness or tiller production) were retained. Articles on less relevant aspects of their genetics (i.e. production of particular enzymes or compounds) were excluded.

Finally, the search term “perennial grain” returned a handful of review papers that addressed the concept of perennial grain systems or utilized it in service of another argument. Reviews that primarily explored perennial grains and their possible utility were retained, while reviews that only mentioned perennial grains in passing were excluded.

We exported the library from Zotero as a CSV file for analysis in R [24]. Separate data frames for each crop were generated by utilizing subset functions in the base R package. We then analyzed the metadata in each data frame to examine trends in publication, highlighting time periods of high publication activity and journals that publish such articles most frequently. Our R code is publicly available via GitHub [25].

### 2.3. Topic modeling

In addition to analyzing bibliographic data, we analyzed publication abstracts using a topic modeling approach. First proposed in 1998 [26], topic modeling in natural language processing is used to identify abstract topics across a dataset. Topics are modeled by analyzing how frequently groups of words co-occur in a given body of literature. Any body of literature may include multiple topics. Much of the literature on perennial staple crops is difficult to synthesize since it comes from disparate sources and fields of scholarship. Topic modeling is well-suited to deal with such a situation.

The workflow that follows was applied to the library for all journal articles, to collections of journal articles for each crop, and to collections of journal articles published in three time periods (1930–1959, 1960–1989, and 1990–2015). We patterned a workflow after Chavalarias and Cointet [27] and Grün and Hornik [28]. We utilized the openNLP R package [29] to tag nouns and adjectives from each document abstract within each crop library. All words were changed to lowercase, and all punctuation, URLs, crop terms, and stop-words were removed. Finally, words were stemmed to remove variable endings using Porter's stemming algorithm [30]. Next, the processed corpus was converted into a document-term matrix, and terms with a sparsity greater than 0.99 were removed.

Topic models with three topics each were then generated using four different methods similar to Grün and Hornik [28]: VEM, VEM-fixed, LDA-Gibbs, and CTM. We chose to model three topics *a priori* since we expected research to generally segregate into the three fields of genetics and breeding, agronomy, and environmental sciences. These three fields represent what we believed might be a hypothetical progression of perennial crop research: breeding to develop a viable crop, agronomic testing to understand performance, and environmental science to test for potential impacts on agroecosystems. We then examined topics by extracting the terms with a likelihood greater than 1% of being associated with a given topic.

For the purposes of this article, we discuss the results of the models using the LDA-Gibbs approach (Latent Dirichlet Allocation and Gibbs sampling). LDA is a generalization of probabilistic latent semantic indexing that allows journal articles to contain a mixture of multiple topics [31]. Gibbs sampling is a Markov Chain Monte Carlo algorithm for obtaining a sequence of observations from a multivariate probability distribution, and is particularly useful for calculating posterior distributions of a Bayesian network [32]. The LDA-Gibbs approach provides posterior probability estimates for how individual terms are assigned to each topic and which journal articles are assigned to each topic. Results were further analyzed by plotting the changes in topic distribution over time and by collectively studying the journal articles that were assigned to each topic.

## 3. Results

### 3.1. Bibliometric analysis

**Fig 2:**
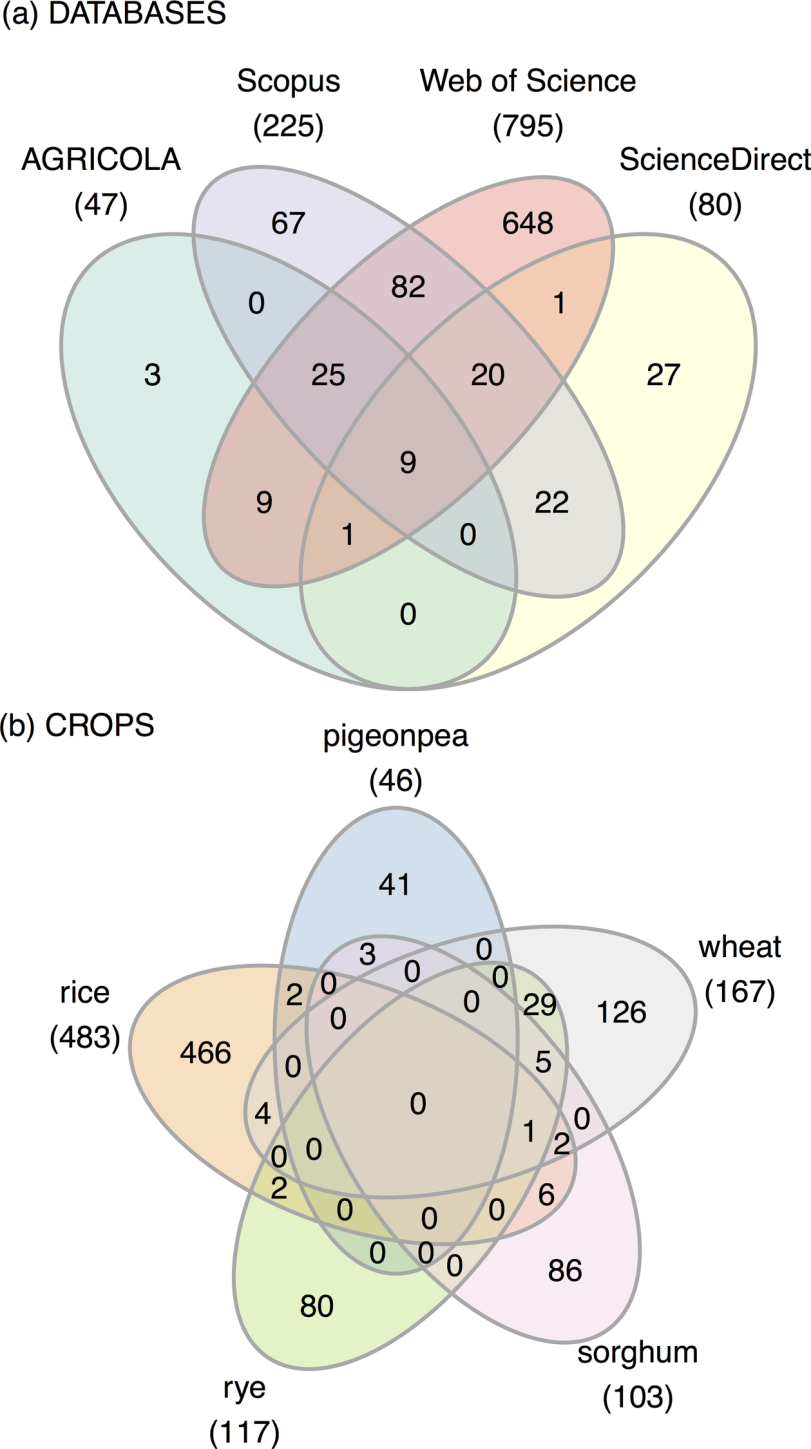
Venn diagrams: of (a) journal articles retrieved from Web of Science (WoS), Scopus, ScienceDirect, and AGRICOLA; and of (b) journal articles queried per crop.

The final library of 914 records was retrieved primarily from WoS, followed by Scopus, ScienceDirect, and Agricola Fig 2A Searches related to perennial rice yielded the most results, followed by wheat, sorghum, rye, and pigeon pea Fig 2B. There was little overlap between crops, with the exception of 29 journal articles that were related to both wheat and rye.

**Fig 3:**
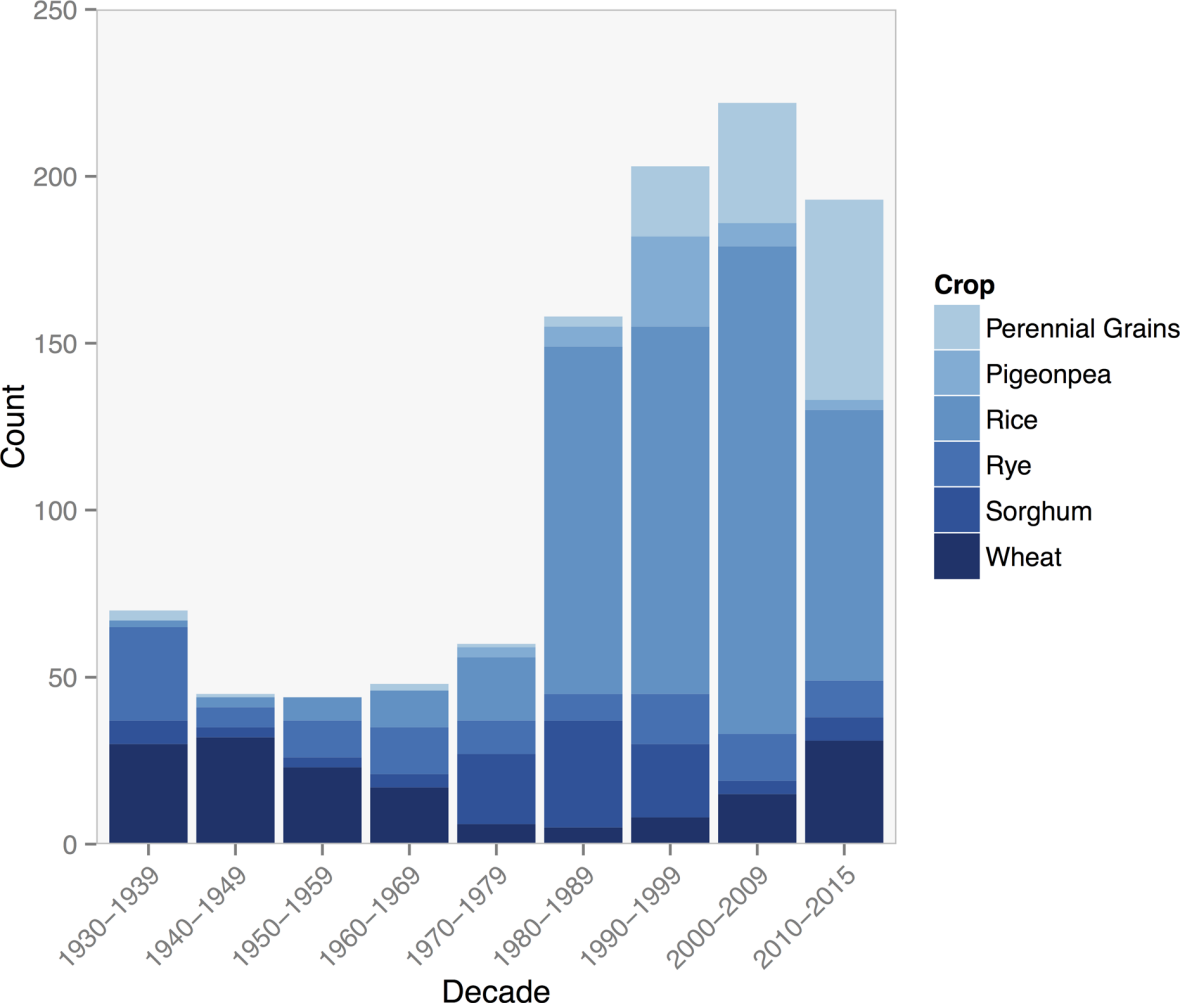
Publication counts per crop by decade, 1930–2015.

Journal articles on perennial crops appeared as early as 1930, and the publication rate since has steadily increased Fig 3. Additionally, the number of journal articles in the last five years from 2010 to 2015 has approached close to the same count from the prior decade (2000–2009). However, the use of the term “perennial grain” has only gained prominence since 2000. Journal articles that use the term appear most frequently in *Agriculture*, *Ecosystems*, *and Environment*, a publication that focuses on the interface between agroecosystems and their environment.

**Fig 4:**
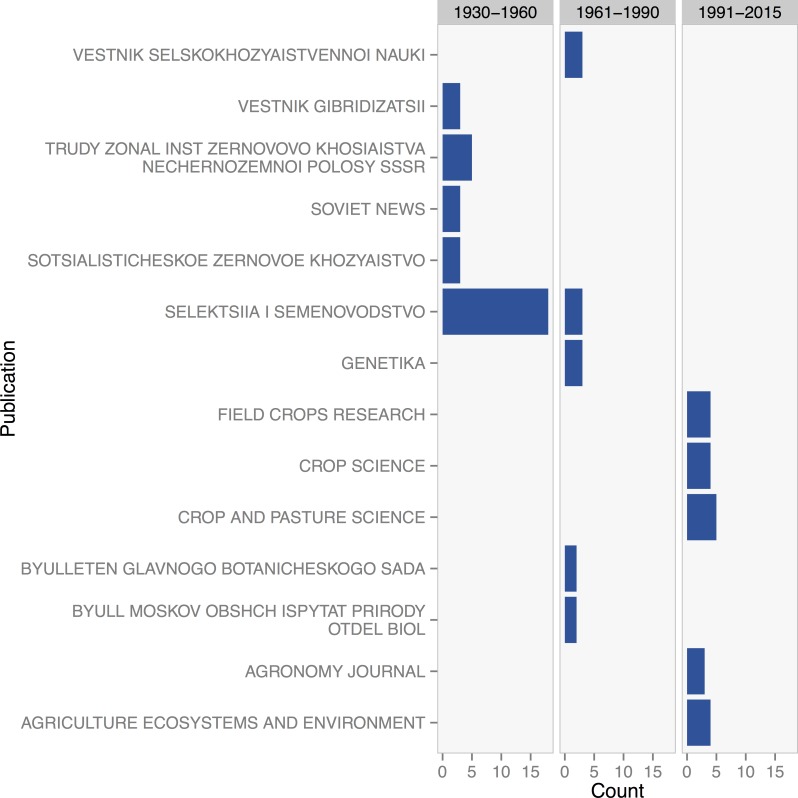
Counts of literature on perennial wheat in the top five publications from the periods of 1930–1960, 1961–1990, and 1991–2015.

Individual crops displayed unique patterns of research activity over time. The early period from 1930 to 1959 were largely represented by perennial wheat research from the former Soviet Union in such publications as *Selektsiya i Semenvodstvo*, which translates to *Breeding and Seed Production* Fig 4. Perennial wheat research decreased between 1960–1989, however in recent years perennial wheat has gained interest in international publications such as *Crop Science*, *Field Crops Research*, *Crop and Pasture Science* and *Agriculture*, *Ecosystems and Environment*.

The period from 1960 to 1989 highlighted publications from India, the Philippines, and the International Rice Research Institute (IRRI), with a focus on perennial rice since the 1980s Fig 5. The majority of journal articles about perennial rice appeared in the International *Rice Research Newsletter*, a publication of IRRI. Indexed, international journals in the agronomy and crop science fields such as *Field Crops Research* began to feature articles on perennial crops research in the 1990s.

**Fig 5:**
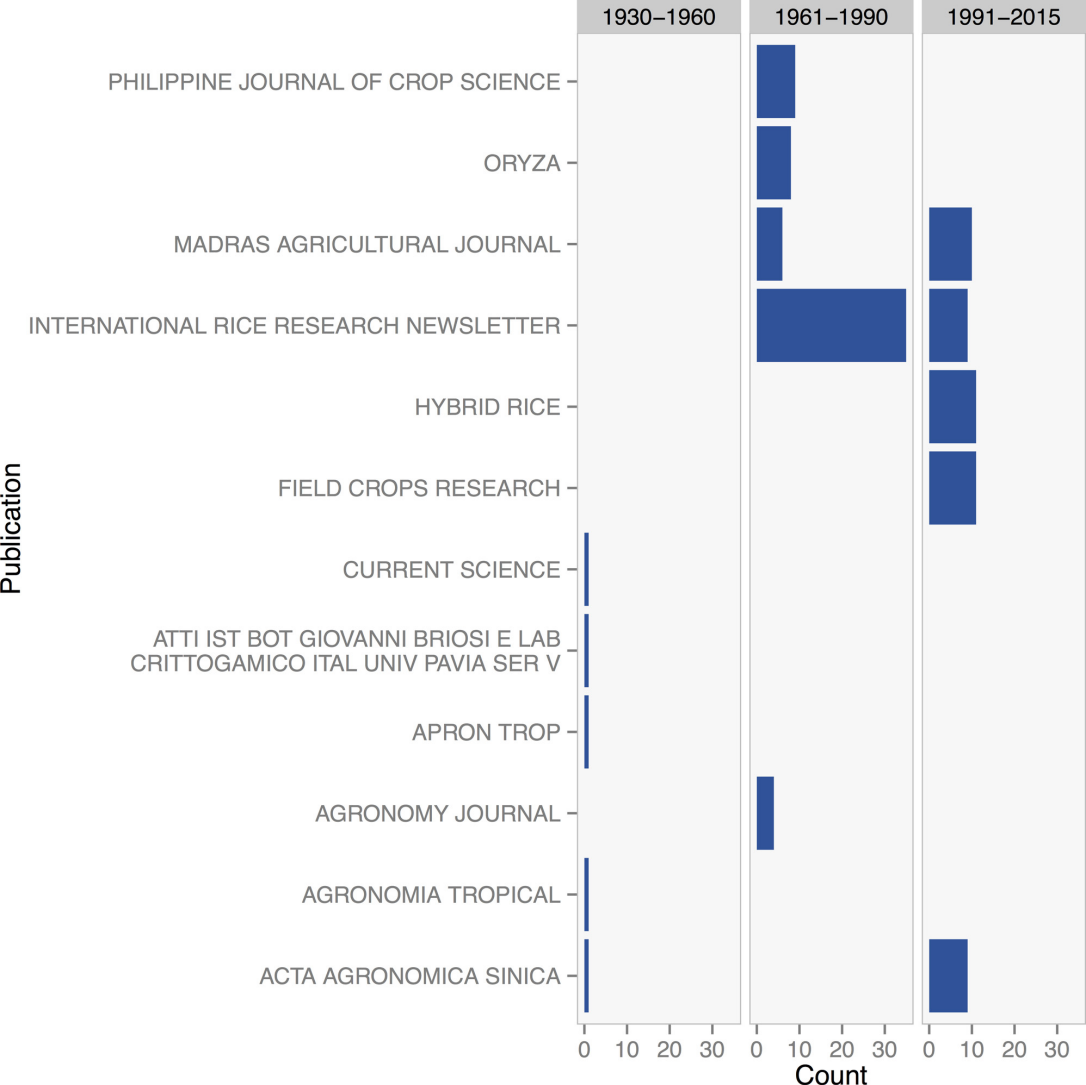
Counts of literature on perennial rice in the top five publications from the periods of 1930–1960, 1961–1990, and 1991–2015.

Perennial pigeon pea and sorghum have received comparatively less attention than the previously mentioned crops, however their rates of publication increased in the 1990s as well (data not shown). Research on perennial pigeon pea was predominantly from India where it is a native crop important to culture and cuisine. Additionally, perennial pigeon pea research has appeared in international publications with foci on agroecology and agroforestry such as *Agroforestry Systems*. Journal articles on perennial sorghum are mostly published in agronomy journals since the 1960s, as well as a few Indian journals such as *Madras Agricultural Journal* and *Journal of Maharashtra Universities*.

### 3.2. Topic modeling

The efficacy of topic models for the library and collections varied considerably. The analysis of the entire library resulted in the highest posterior probabilities for topic terms and journal articles (S1 Appendix; S2 Appendix). The effectiveness of topic modeling for the collections on individual crops was strongly influenced by the size of each collection and the similarity between journal article abstracts. In particular, the pigeon pea collection produced few terms with strong probabilities of association with their topics. However, the two most researched crops, rice and wheat, produced better topic models that included terms and associated articles with higher posterior probabilities and more coherent themes.

Splitting the library by time period had a similar effect to splitting the library into collections by crop. Earlier time periods had many fewer publications compared to the last 30 years, reducing the overall effectiveness of topic modeling. Furthermore, since research within each time period was often dominated by one crop (i.e. 1930–1959 was largely perennial wheat research), splitting the library by time did not necessarily provide further insights beyond the other approaches. Here we present the results of the topic models of the entire library across time and the topic models of the rice and wheat collections as these were most successful in generating multiple coherent topics that matched the papers with high posterior probabilities Table 2.

**Table 2:** Summary of topics generated for each topic modeling approach. For complete lists of terms with high posterior probabilities for each topic see S1 Appendix.

	Topic 1	Topic 2	Topic 3
**Entire library**	*Wheat breeding and rice genetics*: breeding of perennial wheat crops through distant hybridization and rice genome analysis	*Ratoon rice*: physiology and agronomic management of ratoon rice systems	*Agroecology*: interactions between perennial crops and their environments
**Wheat**	*Physiology and impacts*: physiology of perennial crop species and potential impact of perennial crops on agroecosystems	*Breeding*: breeding and agronomy of perennial wheat	*Breeding*: breeding of wheat and rye lines for disease resistance through distant hybridization
**Rice**	*Genetics*: characterizing perennial cultivars and wild relatives	*Ratoon*: nutrient management of ratoon rice and its integration into cropping systems	*Ratoon*: Physiological response of ratoon rice to harvest height, harvest timing, and nitrogen applications

#### 3.2.1. Entire library (1930–2015)

Topic 2 for the entire library includes terms that are primarily about ratoon rice production, such as *ratoon*, *main crop*, and *ratoon crop*. Articles strongly associated with topic 2 are almost exclusively about the physiology and agronomic management of ratoon rice systems. Topic 3 is a more general topic that includes several agronomic terms, such as *plant*, *product*, *crop*, and *soil*. Topic 3 articles generally discuss interactions between perennial crops and their environment. Topic 1 is comprised of terms that refer to the breeding of perennial crops through hybridization of distantly related species, such as *perennial*, *annual*, *hybrid*, and *resist*. Strongly associated articles with topic 1 are primarily from the early Soviet literature in the 1930s.

**Fig 6:**
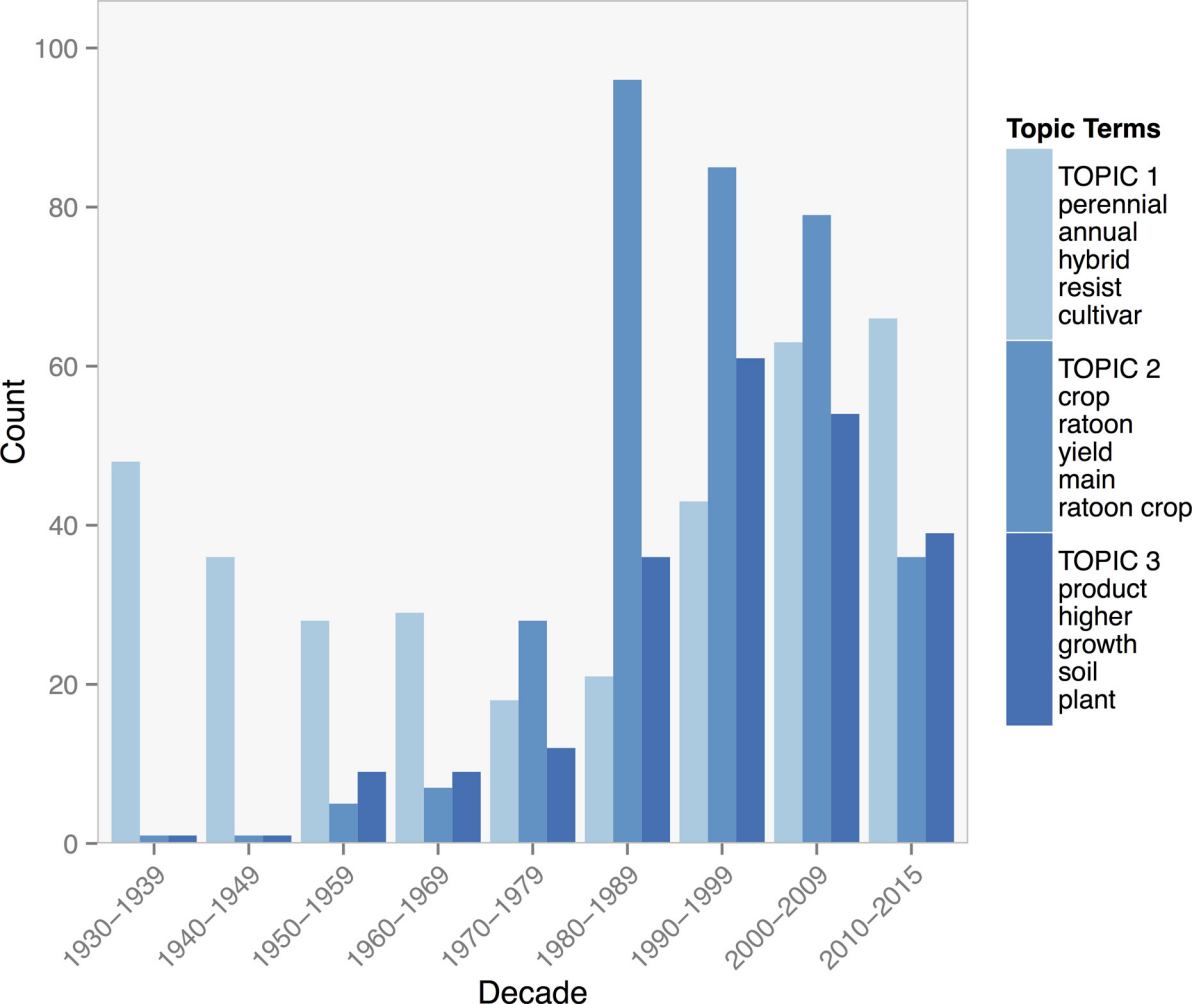
Counts of journal articles assigned by the Gibbs model of the entire library to each of three topics by decade. The five topic terms with the highest posterior probability of association with each topic are listed with their corresponding topic in the legend.

The distribution over time of topics 3 and 1 matched the publication patterns for wheat and rice outlined above Fig 6. Topic 3 had high counts in the early twentieth century when research on the breeding of perennial wheat appeared frequently in Soviet journals, decreased in the middle of the century, but has more recently seen an increase. Topic 1 had a low publication count until about the 1980s when research on ratooning rice systems dramatically increased and was sustained until about 2010. Topic 2 on crop by environment interactions steadily increased throughout the 20th century with a particularly large increase since the 1980s.

#### 3.2.2. Wheat collection (1930–2015)

Topic terms for the wheat collection largely reflected the fields of breeding and agronomy on wheat. Topic 2 included general terms, such as *perennial*, *type*, *number* and *fertility*, as well as the genus name *agropyron* (*Agropyron glaucum*) and the species name *elongatum* (*Thinopyrum elongatum*). These two species were used by breeders in the Soviet Union to introduce perennial characteristics into their breeding lines. Articles associated with topic 2 reflect these terms in that they cover the selection, phenology, and physiology of those lines. Terms for topic 3 were more generally about distant hybridization, including terms such as *hybrid*, *variety*, and *cross*. Topic 1 included a broad range of general agronomic terms such as *perennial*, *annual*, *crop*, *yield*, and *soil*. Despite how broad these terms were, the articles created a cohesive group focused on various aspects of the physiology of perennial plants and their potential implications for agronomic performance.

**Fig 7:**
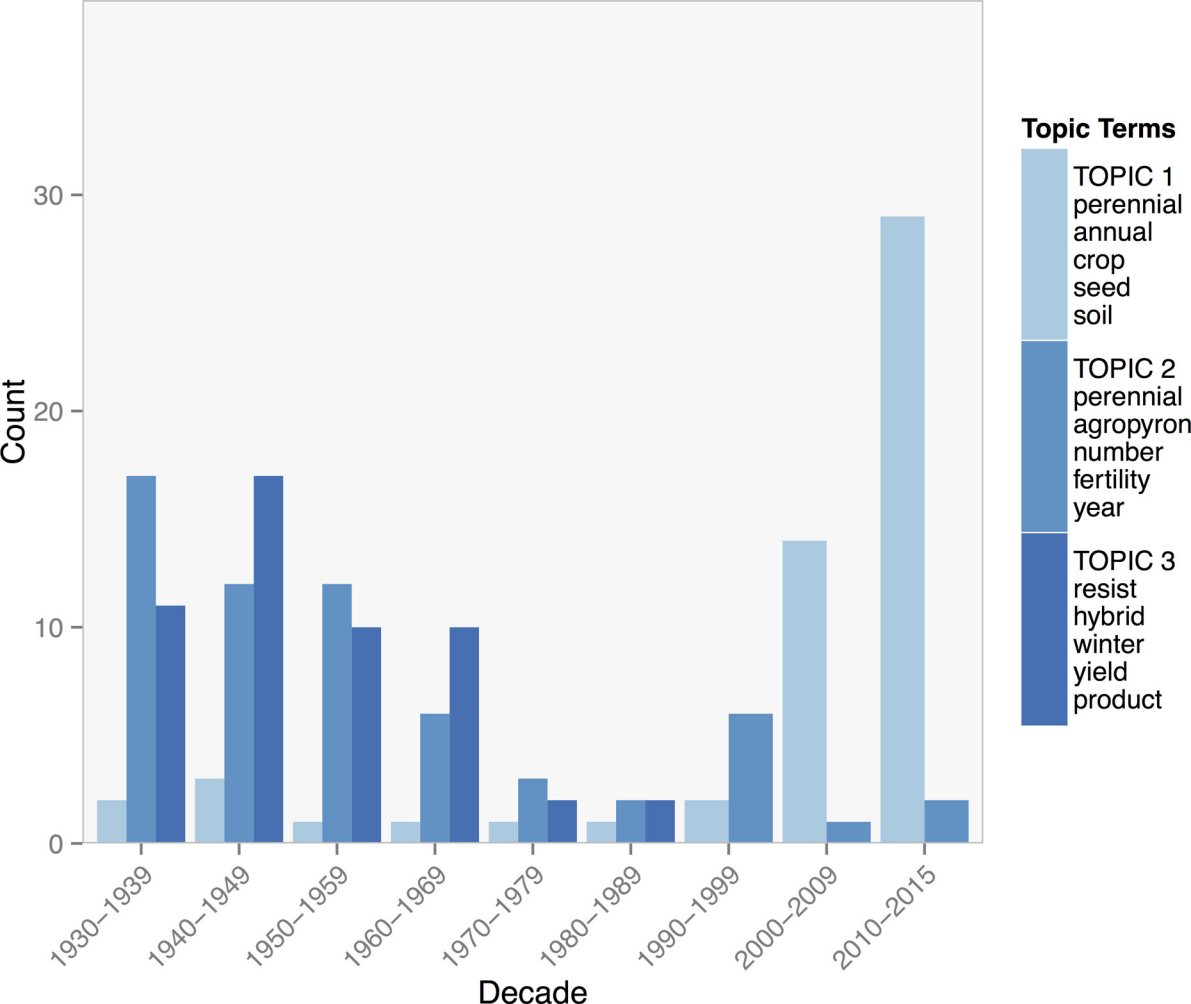
Counts of journal articles assigned by the Gibbs model of the wheat collection to each of three topics by decade. The five topic terms with the highest posterior probability of association with with their corresponding topic in the legend.

The distribution of topics over time for the wheat collection reflected the bimodal pattern of research on perennial wheat Fig 7. Topics 2 and 3 were most strongly associated with the breeding of perennial wheat in the USSR between 1930 to 1970 that was motivated by Soviet expeditions to centers of diversity for domesticated crops. Topic 1 on the physiology of perennial wheat, among other perennial species, and its potential impacts on agroecosystems were most present in recent research from the 2000s onward.

#### 3.3.3. Rice collection (1930–2015)

Both topics 2 and 3 for the collection on perennial rice were concerned with ratoon systems with the term *ratoon* high on the list of probable terms. The other terms for either topic emphasize different aspects of ratooning systems. Topic 2 includes general terms such as *yield*, *main crop*, *product*, and *field*, while topic 3 includes such terms as *height*, *stubble*, *panicle*, and *nitrogen*. Journal articles associated with topic 2 were focused on the effects of nutrient management on ratoon rice, and its integration into cropping systems, while journal articles strongly associated with topic 3 were primarily about the physiological response of ratoon rice to harvest height, timing, and nitrogen applications. Topic 1, on the other hand, was concerned with characterizing perennial cultivars and wild relatives, with terms including *perennial*, *population*, and *genetic*.

All topics saw a steady increase over the past century with a large jump in the number of publications in the 1980s Fig 8. However by the 2000s, topic 3 on panicle production and grain filling of ratooned rice declined. Topic 2 on the agronomic management of ratoon crops and topic1 on the phylogeny and genetics of rice have continued to increase.

**Fig 8:**
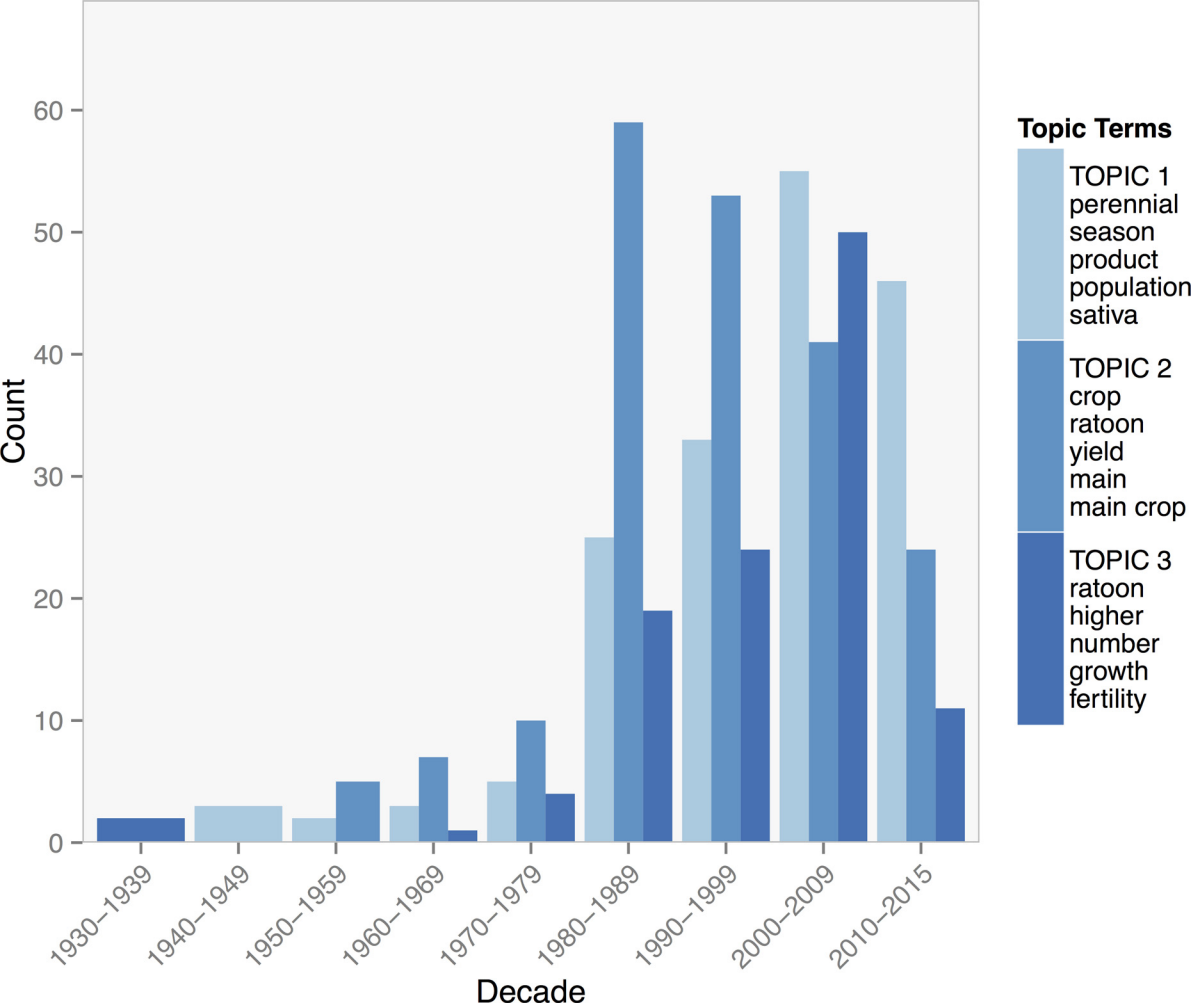
Counts of journal articles assigned by the Gibbs model of the rice collection to each of three topics by decade. The five topic terms with the highest posterior probability of association with with their corresponding topic in the legend.

## 4. Discussion

### 4.1. Bibliometric analysis

Overlaps in research between some crops are likely due to their agronomic compatibility or genetic relatedness. For example, literature on wheat and rye often overlapped since they are closely genetically related and were both used as source material in various perennial crop breeding efforts.

While research on perennial crops is increasing overall, there appear to have been distinct periods of active research for each crop. We observed that research on perennial crops has generally transitioned from exploratory phases to eventually gain prominence through publications with a broader readership. At the exploratory, initial phase, dialog was primarily confined within small groups and specific communities of researchers, with articles primarily appearing in special interest or crop-specific publications. Rice provides a clear example, where the publication of research during the 1980s and 1990s was led by IRRI, and primarily consisted of newsletter and technical articles, followed later by the appearance of articles in more broadly circulated crop physiology journals. Likewise, journal articles on perennial wheat were primarily from the Soviet Union in the early 20th century and published in Russian language journals, while the recent increase in publication over the past 10 years has been primarily in prominent agronomy and agroecology journals.

Certain publications have played an important role in fostering particular research areas. *Field Crops Research* and *Agriculture*, *Ecosystems*, *and Environment* emerge as primary publishers for the crops evaluated and for the more general topic of perennial grains. Both publications have impact factors around 3, and are top journals in their research areas. Further, they are often target publications for the fields of agronomy and breeding. The consistency with which such journals have articles on perennial crops in recent decades may indicate a favorable editorial environment that may help advance progress in perennial crops research.

### 4.2. Topic modeling

Contrary to our hypothesis, topic models for the entire library did not sort out into the three fields of research we expected—breeding, agronomy, and agroecology. Instead the higher numbers of rice and wheat publications guided topic models of the full library, generating topic terms that were highly specific to either crop and the approach researchers took to developing their perenniality or ratooning characteristics for agronomic purposes. Perennial crops research in the early 20th century was dominated by USSR breeding efforts and their technique of distant hybridization (i.e. topic 1). While research on rice was primarily about the physiology and management of ratoon rice systems (i.e. topic 2). Topic models of the rice and wheat collections also did not produce topics that matched our hypothesized categories.

Topic 3 from the topic model of the entire library is the only topic that covered a field similar to our hypothesized category on agroecology. Papers associated with this topic included research on pest dynamics, soil moisture dynamics, and greenhouse gas budgets. Overall, however, the almost complete lack of topics dealing explicitly with soil biology and soil carbon is notable since some of the most prominent arguments for perennial cropping systems posit dramatic improvements in soil quality and ecological functions in agricultural landscapes [1]. Environmental traits of perennial crops such as massive production of roots and biomass to support soil C gains and retention of nitrogen for water quality have been described in high profile papers [4,33]. Yet there are very few perennial crop papers that report primary data on soil related topics. This may be due to the fact that there are few cultivars that have been released that exhibit true perenniality. Germplasm is still under development, few lines in perennial wheat for example are stable and many have undesirable traits [34]. Perennial crops researchers, then, are primarily engaged in developing cultivars for better perenniality and agronomic traits rather than researching the agroecology of the systems in which they are grown.

This bias in the literature toward developing crops that achieve both high yield and high annual survival may be coming at the expense of research that better reflects growers’ motivations for choosing such crops. Adebiyi et al. [5] found in interviews with farmers that improvements to soil and environmental quality were primary motivators for their interest in perennial wheat and that achieving yields competitive with annual wheat were of secondary importance. Higher yields are important to industrial cropping system models where commercial output and financial risk avoidance are paramount. But perennial crops may be better suited to alternative cropping system models or secondary producer objectives, meaning the overwhelming focus on agronomy and breeding is partially misplaced.

Topic modeling for the rice and wheat collections reveal differences in the patterns of research on these two crops that highlight interesting differences in strategies to develop and exploit perenniality. The comparatively large number of publications on rice is largely due to the inclusion of literature on ratoon rice systems, and topic models were dominated by different aspects of ratoon system research. The importance of rice ratooning should not be understated, as it is a system often practiced in different rice growing regions of the world in both commercial and smallholder contexts. Institutional efforts to support the development of ratoon systems are apparent in the large number of publications on rice that appear in the International Rice Research Newsletter, a publication of IRRI. Although ratoon rice systems are not perennial in the sense that they last for several years, the existence of such a broadly practiced, traditional system has arguably provided support and a starting point for institutional breeding efforts. Recent research at the Yunnan Agricultural University in China has produced a variety of rice that successfully ratoons from year to year [14,15].

Literature on wheat, on the other hand, was dominated by breeding efforts until much more recently when research topics on the physiology of perennial wheat cultivars and the agroecological potential of such systems appear to emerge. A practice analogous to ratooning rice arguably does not exist for wheat. Lacking a similar system to utilize as a starting point, efforts to breed perennial wheat may have been more handicapped. Breeding efforts in the Soviet Union required hybridizing wheat with more distantly related species, such as *Agropyron glaucum*. Decades of effort managed to produce some promising lines, but only after considerable difficulty with issues of sterility, low annual survival, and unfavorable agronomic characteristics [10,35]. Similarly, more recent efforts have produced lines with strong perenniality but underdeveloped agronomic characteristics and yield potential [36]. Despite such obstacles, perennial wheat breeders argue it still has strong potential and may simply require more extended breeding efforts targeted at selection of particular suites of traits [37]. Or, as McClure, et al. [38] argue, perennial crop breeding may be accelerated by modern genomics techniques. The emergence of topic 3 for the topic model of the wheat collection, which includes terms and papers concerned with the physiology of perennial wheat, may also indicate a transition in research strategy towards a more basic understanding of source/sink limitations on the perenniality of perennial wheat cultivars.

The comparison of rice and wheat also serves to highlight potential gaps in perennial crop breeding efforts with other crops. If a foundation of research on ratooning rice systems has provided a comparative advantage for developing successful perennial cultivars of rice, other crops, such as pigeon pea and sorghum, that have been similarly utilized as ratooning crops in traditional systems, may be primed for development, Although we included them in our searches, fewer publications for pigeon pea and sorghum were retrieved despite their historic and continued use as perennials in smallholder production systems. Pigeon pea originated as a perennial species and is still used as such in agroforestry systems, and is a widespread garden species in tropical gardens; however breeding for short duration varieties is the dominant trend in pigeon pea crop development. Sorghum is managed as a ratoon crop in some regions of Africa, however its documentation and development in the scientific literature is limited. This lack of publications is likely due various causes, but is perhaps best explained by it being primarily produced under semi-arid and marginal soil conditions. The same bias towards developing high yielding, commercially relevant perennial crops discussed above might explain the lack of interest in sorghum and pigeonpea among researchers. If greater effort were focused on developing these crops, existing knowledge systems and varieties could be effectively leveraged to generate truly perennial lines as appears to be the case with rice.

## 5. Conclusions

Analysis of perennial crops publications has highlighted interesting research development trajectories and publishing patterns over the past century. Each crop has seen episodic waves of interest with higher publication rates, but some crops are considerably less researched. The dominance of certain journals in the libraries indicates the importance of individual journals to supporting a field of research, especially journals that are crop-specific, such as the *International Rice Research Newsletter*. Topic modeling of the entire library was strongly influenced by the high numbers of rice and wheat publications but also produced a topic about the agroecology of perennial and ratooning systems. No topics explicitly concerning soil biology or carbon dynamics were produced, reflecting a dearth of publications in this area and potential bias amongst researchers. A comparison of topic models for rice and wheat revealed differences between their histories of development. The existence and widespread use of ratoon rice systems has provided source material for developing perennial rice cultivars, while the lack of an analogous system for wheat has meant breeding efforts had limited material and research on which to base their research. Comparatively little has been done to develop some other perennial crops, such as pigeon pea and sorghum. But their use as perennials in some contexts may make them prime candidates for such development. However whether they receive such attention may depend on the priorities of researchers and the development sector.

## Supporting Information

S1 Appendix Tables containing topic terms that have at least a 1% posterior probability of association with topics produced by corresponding Gibbs topic model. (DOCX)

S2 Appendix Tables containing citation information for articles strongly associated with topics produced by corresponding Gibbs topic model. (DOCX)

S1 PRISMA Checklist PRISMA Checklist. Completed PRISMA checklist for meta-analyses.
(DOC)
