# Supplementary material for: A Systematic Review of Perennial Staple Crops Literature Using Topic Modeling and Bibliometric Analysis
Source: PLoS One. 2016 May 23;11(5):e0155788. doi: 10.1371/journal.pone.0155788 (PMC4877017; doi:10.1371/journal.pone.0155788)
Supplement: S1 Appendix — (DOCX) [file pone.0155788.s001.docx]

**Table A**.

| **Topic 1** | | **Topic 2** | | **Topic 3** | |
| --- | --- | --- | --- | --- | --- |
| *Term* | *Probability* | *Term* | *Probability* | *Term* | *Probability* |
| perennial | 0.0744 | crop | 0.0966 | product | 0.025 |
| annual | 0.0222 | ratoon | 0.0932 | higher | 0.0241 |
| hybrid | 0.0208 | yield | 0.069 | growth | 0.0231 |
| resist | 0.0173 | main | 0.0351 | soil | 0.0219 |
| cultivar | 0.0166 | ratoon crop | 0.0245 | plant | 0.0212 |
| wild | 0.0161 | main crop | 0.0235 | high | 0.0165 |
| winter | 0.0137 | harvest | 0.022 | field | 0.0163 |
| genetic | 0.0108 | fertility | 0.0192 | season | 0.0162 |
|  |  | number | 0.0184 | rate | 0.0157 |
|  |  | height | 0.0158 | leaf | 0.015 |
|  |  | highest | 0.0157 | drier | 0.0146 |
|  |  | effect | 0.0149 | population | 0.0146 |
|  |  | ability | 0.0148 | stage | 0.0143 |
|  |  | plant | 0.0133 | develop | 0.0135 |
|  |  | significant | 0.0121 | low | 0.0124 |
|  |  | weight | 0.0111 | water | 0.0123 |
|  |  | panicle | 0.0106 | earlier | 0.0108 |
|  |  | mature | 0.0103 | lower | 0.0108 |
|  |  | stubble | 0.0101 | seed | 0.0105 |

Topic terms that have at least a 1% posterior probability of association with topics according to the Gibbs model for the entire library (1930-2015).

**Table B.**

| **Topic 1** | | **Topic 2** | | **Topic 3** | |
| --- | --- | --- | --- | --- | --- |
| *Term* | *Probability* | *Term* | *Probability* | *Term* | *Probability* |
| perennial | 0.0655 | perennial | 0.067 | resist | 0.0391 |
| annual | 0.0295 | agropyron | 0.031 | hybrid | 0.0295 |
| crop | 0.0207 | number | 0.0174 | winter | 0.0242 |
| seed | 0.0167 | fertility | 0.0168 | yield | 0.0188 |
| soil | 0.0167 | year | 0.0146 | product | 0.0138 |
| yield | 0.0167 | elongatum | 0.0118 | cultivar | 0.0129 |
| plant | 0.0112 | spring | 0.0115 | select | 0.0127 |
| develop | 0.0109 | type | 0.0109 | varieties | 0.0127 |
|  |  |  |  | wild | 0.0127 |
|  |  |  |  | cross | 0.0124 |
|  |  |  |  | work | 0.0115 |
|  |  |  |  | high | 0.0101 |

Topic terms that have at least a 1% posterior probability of association with topics according to the Gibbs model for the wheat collection (1930-2015).

**Table C.**

| **Topic 1** | | **Topic 2** | | **Topic 3** | |
| --- | --- | --- | --- | --- | --- |
| *Term* | *Probability* | *Term* | *Probability* | *Term* | *Probability* |
| perennial | 0.022 | crop | 0.1091 | ratoon | 0.0546 |
| season | 0.0217 | ratoon | 0.0734 | higher | 0.027 |
| product | 0.0195 | yield | 0.0618 | number | 0.0237 |
| population | 0.0171 | main | 0.048 | growth | 0.0228 |
| sativa | 0.0134 | main crop | 0.0353 | fertility | 0.0214 |
| wild | 0.0131 | ratoon crop | 0.03 | ability | 0.0203 |
| cultivar | 0.013 | harvest | 0.0272 | rate | 0.0179 |
| develop | 0.013 | plant | 0.0182 | significant | 0.0172 |
| studies | 0.0129 | highest | 0.0164 | stage | 0.0171 |
| plant | 0.0116 | height | 0.0138 | panicle | 0.0166 |
| water | 0.0111 | field | 0.012 | drier | 0.0159 |
| low | 0.0109 | effect | 0.0112 | stubble | 0.0147 |
| annual | 0.0107 | soil | 0.0101 | total | 0.0143 |
| genetic | 0.0104 |  |  | leaf | 0.0137 |
|  |  |  |  | application | 0.0136 |
|  |  |  |  | weight | 0.0135 |
|  |  |  |  | high | 0.0125 |
|  |  |  |  | nitrogen | 0.012 |
|  |  |  |  | mature | 0.011 |
|  |  |  |  | yield | 0.0107 |
|  |  |  |  | hybrid | 0.0102 |

Topic terms that have at least a 1% posterior probability of association with topics according to the Gibbs model for the rice collection (1930-2015).
