# Supplementary material for: A Systematic Review of Perennial Staple Crops Literature Using Topic Modeling and Bibliometric Analysis
Source: PLoS One. 2016 May 23;11(5):e0155788. doi: 10.1371/journal.pone.0155788 (PMC4877017; doi:10.1371/journal.pone.0155788)
Supplement: S2 Appendix — (DOCX) [file pone.0155788.s002.docx]

**Table A.**

| **Topic** | **Article** | **Probability** |
| --- | --- | --- |
| 1 | Firsova, M., 1948. Research Institutes in struggles for harvests. Sel. Semenovod. 15, 66–72. | 0.764 |
|  | Vakar, B., 1935. Wheat-Agropyrum hybrids: a hylogenetic investigation. Tr. Prikl Bot Genet Sel. Ser 2, 121–61. | 0.6938 |
|  | Petit, J., Bourgeois, E., Stenger, W., Bès, M., Droc, G., Meynard, D., Courtois, B., Ghesquière, A., Sabot, F., Panaud, O., Guiderdoni, E., 2009. Diversity of the Ty-1 copia retrotransposon Tos17 in rice (Oryza sativa L.) and the AA genome of the Oryza genus. Mol. Genet. Genomics 282, 633–652. doi:10.1007/s00438-009-0493-z. | 0.6853 |
|  | Prutskova, M., 1934. Breeding grain crops. Bull Appl Bot Leningr. Ser A 93–103. | 0.6808 |
|  | Gopala, K.S., Waters, D.L.E., Henry, R.J., 2014. Australian wild rice reveals pre-domestication origin of polymorphism deserts in rice genome. PLoS ONE 9. doi:10.1371/journal.pone.0098843. | 0.6787 |
| 2 | Huossainzade, A., Azarpour, E., Doustan, H.Z., Moraditochaee, M., Bozorgi, H.R., 2011. Management of cutting height and nitrogen fertilizer rates on grain yield and several attributes of ratoon rice (Oryza sativa L.) In Iran. World Appl. Sci. J. 15, 1089–1094. | 0.7495 |
|  | Chauhan, J.S., Lopez, F.S.S., Vergara, B.S., 1985. Effect of harvest time on IR44 ratoon grain yield. Int. Rice Res. Newsl. 10, 26–27. | 0.7222 |
|  | Daliri, M.S., Eftekari, A., Mobasser, H.R., Tari, D.B., Porkalhor, H., 2009. Effect of cutting time and cutting height on yield and yield components of ratoon rice (Tarom Langrodi Variety). Asian J. Plant Sci. 8, 89–91. doi:10.3923/ajps.2009.89.91. | 0.7167 |
|  | Turner, F., Jund, M., 1993. Rice ratoon crop yield linked to main crop stem carbohydrates. Crop Sci. 33,150-153. | 0.706 |
|  | Yazdpour, H., Shahri, M.M., Soleymani, A., Shahrajabian, M.H., 2012. Effects of harvesting time and harvesting height on grain yield and agronomical characters in rice ratoon (Oryza sativa L.). J. Food Agric. Environ. 10, 438–440. | 0.6938 |
| 3 | Cheng, C.H., Huang, S.H., 2004. Population fluctuations and forecasting of the white-backed planthopper, Sogatella furcifera on rice in Chiayi region, Taiwan. Plant Prot. Bull. Taichung 46, 315–332. | 0.6908 |
|  | Hulugalle, N.R., Lal, R., 1986. Root growth of maize in a compacted gravelly tropical alfisol as affected by rotation with a woody perennial. Field Crops Res. 13, 33–44. doi:10.1016/0378-4290(86)90005-5. | 0.6373 |
|  | Alberto, M.C.R., Hirano, T., Miyata, A., Wassmann, R., Kumar, A., Padre, A., Amante, M., 2012. Influence of climate variability on seasonal and interannual variations of ecosystem CO2 exchange in flooded and non-flooded rice fields in the Philippines. Field Crops Res. 134, 80–94. doi:10.1016/j.fcr.2012.05.002. | 0.6366 |
|  | Piper, J. k., 1993. Soil water and nutrient change in stands of three perennial crops. Soil Sci. Soc. Am. 57, 497–505. | 0.6202 |
|  | González-Paleo, L., Ravetta, D., 2011. Relationships between reproductive output, morpho-physiological traits and life span in Lesquerella (Brassicaceae). Ind. Crops Prod. 34, 1386–1392. doi:10.1016/j.indcrop.2011.02.001. | 0.6085 |

Journal articles associated with Gibbs model topics for entire library (1930-2015).

**Table B.**

| **Topic** | **Article** | **Probability** |
| --- | --- | --- |
| 1 | Jaikumar, N.S., Snapp, S.S., Flore, J.A., Loescher, W., 2014. Photosynthetic Responses in Annual Rye, Perennial Wheat, and Perennial Rye Subjected to Modest Source: Sink Ratio Changes. Crop Sci. 54, 274–283. doi:10.2135/cropsci2013.04.0280. | 0.7192 |
|  | Ploschuk, E.L., Slafer, G.A., Ravetta, D.A., 2005. Reproductive allocation of biomass and nitrogen in annual and perennial Lesquerella crops. Ann. Bot. 96, 127–135. doi:10.1093/aob/mci158. | 0.7096 |
|  | Zhang, Y., Li, Y., Jiang, L., Tian, C., Li, J., Xiao, Z., 2011. Potential of Perennial Crop on Environmental Sustainability of Agriculture. Procedia Environ. Sci. 10, Part B, 1141–1147. doi:10.1016/j.proenv.2011.09.182. | 0.708 |
|  | González-Paleo, L., Ravetta, D., 2011. Relationships between reproductive output, morpho-physiological traits and life span in Lesquerella (Brassicaceae). Ind. Crops Prod. 34, 1386–1392. doi:10.1016/j.indcrop.2011.02.001. | 0.6909 |
|  | Dawson, J.C., Huggins, D.R., Jones, S.S., 2008. Characterizing nitrogen use efficiency in natural and agricultural ecosystems to improve the performance of cereal crops in low-input and organic agricultural systems. Field Crops Res. 107, 89–101. doi:10.1016/j.fcr.2008.01.001. | 0.6799 |
| 2 | Cicin, N.V., 1936. The problem of perennial wheat. Sel. Semenovod. 21–7. | 0.6884 |
|  | Schneiderman, J., 1946. Forms of perennial and autumn tillering wheat. Social. Zernovoe Hozjaisivo Social. Grain Farming 145–51. | 0.6459 |
|  | Hiznjak, V.A., 1936. A cyto-genetical study of Triticum X Agropyron hybrids and the trend in breeding perennial wheat. Preliminary communication. Tr. Azovo-Cernomor Sel.-Cent 25–30. | 0.6366 |
|  | Derzavin, A., 1936. The problem of perenniality in agricultural plants. Sel. Semenovod. 9–10. | 0.6224 |
|  | Lyubimova, V. f., 1991. Mechanism of incorporation of separate couch grass genomes into the genome complex of durum wheat. Sov. Genet. 27, 717–728. | 0.5671 |
| 3 | Prutskova, M., 1934. Breeding grain crops. Bull Appl Bot Leningr. Ser A 93–103. | 0.6991 |
|  | Firsova, M., 1948. Research Institutes in struggles for harvests. Sel. Semenovod. 15, 66–72. | 0.6625 |
|  | Aldanov, A.D., 1936. 25 years work of Saratov Central Station of plant-breeding and genetics. Sotsialisticheskoe Zernovoe Khozyaistvo 6, 131–42. | 0.659 |
|  | Grigor’eva, N.M., Kuz’min, Z.E., Lanina, A.V., 1968. An All-Union meeting on distant hybridization in plants and animals. Vestn. Selsko-Khozyaistvennoi Nauki 101–10. | 0.6519 |
|  | Vavilov, N.I., 1934. Soviet scientific plant industry during the period of Soviet reconstruction 1930-1933. Bull Appl Bot Leningr. Ser A 5–23. | 0.6309 |

Journal articles associated with Gibbs model topics for wheat collection (1930-2015).

**Table C.**

| **Topic** | **Article** | **Probability** |
| --- | --- | --- |
| 1 | Zhu, Q., Zheng, X., Luo, J., Gaut, B.S., Ge, S., 2007. Multilocus analysis of nucleotide variation of Oryza sativa and its wild relatives: Severe bottleneck during domestication of rice. Mol. Biol. Evol. 24, 875–888. doi:10.1093/molbev/msm005. | 0.7295 |
|  | Gopala, K.S., Waters, D.L.E., Henry, R.J., 2014. Australian wild rice reveals pre-domestication origin of polymorphism deserts in rice genome. PLoS ONE 9. doi:10.1371/journal.pone.0098843. | 0.7214 |
|  | Petit, J., Bourgeois, E., Stenger, W., Bès, M., Droc, G., Meynard, D., Courtois, B., Ghesquière, A., Sabot, F., Panaud, O., Guiderdoni, E., 2009. Diversity of the Ty-1 copia retrotransposon Tos17 in rice (Oryza sativa L.) and the AA genome of the Oryza genus. Mol. Genet. Genomics 282, 633–652. doi:10.1007/s00438-009-0493-z. | 0.7051 |
|  | Uga, Y., Fukuta, Y., Ohsawa, R., Fujimura, T., 2003. Variations of Floral Traits in Asian Cultivated Rice (Oryza sativa L.) and its Wild Relatives (O. rufipogon Griff.). Breed. Sci. 53, 345–352. doi:10.1270/jsbbs.53.345. | 0.7028 |
|  | Grillo, M.A., Li, C., Fowlkes, A.M., Briggeman, T.M., Zhou, A., Schemske, D.W., Sang, T., 2009. Genetic architecture for the adaptive origin of annual wild rice, Oryza nivara. Evolution 63, 870–883. doi:10.1111/j.1558-5646.2008.00602.x. | 0.6932 |
| 2 | Chauhan, J.S., Lopez, F.S.S., Vergara, B.S., 1985. Effect of harvest time on IR44 ratoon grain yield. Int. Rice Res. Newsl. 10, 26–27. | 0.6747 |
|  | Shamiul Islam, M., Hasanuzzaman, M., Rokonuzzaman, M., 2008. Ratoon rice response to different fertilizer doses in irrigated condition. Agric. Conspec. Sci. 73, 197–202. | 0.6667 |
|  | Jones, D.B., 1993. Rice ratoon response to main crop harvest cutting height. Agron. J. 85, 1139–1142. | 0.6506 |
|  | NengPu, S., Zhi, C., YingRi, C., GuangHan, X., ShiLiao, L., 1998. Cultivation techniques of a ratoon crop from broadcast transplanted rice. China Rice 18–19. | 0.6478 |
|  | Yazdpour, H., Shahri, M.M., Soleymani, A., Shahrajabian, M.H., 2012. Effects of harvesting time and harvesting height on grain yield and agronomical characters in rice ratoon (Oryza sativa L.). J. Food Agric. Environ. 10, 438–440. | 0.645 |
| 3 | Nakano, H., Morita, S., 2008. Effects of time of first harvest, total amount of nitrogen, and nitrogen application method on total dry matter yield in twice harvesting of rice. Field Crops Res. 105, 40–47. doi:10.1016/j.fcr.2007.07.002. | 0.737 |
|  | ZhenXie, Y., NaiMei, T., Pu, W., PingPing, C., 2005. Ratooning properties of axillary buds of two-line hybrid rice in vivo and in vitro. Acta Agron. Sin. 31, 330–336. | 0.7259 |
|  | Nakano, H., Morita, S., 2007. Effects of twice harvesting on total dry matter yield of rice. Field Crops Res. 101, 269–275. doi:10.1016/j.fcr.2006.12.001. | 0.6932 |
|  | HongFei, C., ZhiXing, Z., WenXiong, L., 2014. Effects of nitrogen application for bud development on protein expression of ratooning buds of rice. Zhongguo Shengtai Nongye Xuebao Chin. J. Eco-Agric. 22, 1405–1413. | 0.6891 |
|  | Qiang, H., LiYun, C., GuoHua, L., Feng, L., 2004. On correlation between physiological function of root system and ratooning ability of hybrid rice. J. Hunan Agric. Univ. 30, 95–99. | 0.6582 |

Journal articles associated with Gibbs model topics for rice collection (1930-2015).
